# Choosing wisely in neuroradiology: evaluating CT and MR utilization trends in Europe

**DOI:** 10.1007/s00330-026-12528-1

**Published:** 2026-05-12

**Authors:** Conor Brosnan, Andrea Rossi, Anouk van der Hoorn, Paulina Due-Tønnessen, Seamus Looby

**Affiliations:** 1 Department of Neuroradiology, Beaumont Hospital, Dublin, Ireland; 2 Neuroradiology Unit, IRCCS Istituto Giannina Gaslini, Genoa, Italy; 3 Department of Health Sciences (DISSAL), University of Genoa, Genoa, Italy; 4 Department of Radiology, University Medical Center Groningen, University of Groningen, Groningen, The Netherlands; 5 Department of Radiology and Nuclear Medicine, University of Oslo, Oslo University Hospital, Oslo, Norway

**Keywords:** Europe, Magnetic resonance imaging, Neuroimaging, Tomography (X-ray computed), Utilization

## Abstract

**Objectives:**

To assess trends in volume and utilization of neuroimaging (CT and MR) across Europe over the last decade, within the context of evolving clinical practice on behalf of the European Society of Neuroradiology’s Choosing Wisely committee.

**Materials and methods:**

A systematic search of PubMed was performed (following PRISMA 2020 guidelines) to identify studies reporting European neuroimaging volumes. As no eligible studies were identified, descriptive analysis of Eurostat and OECD data was performed for 29 European countries from 2015 to 2022, covering CT and MR examination volumes and scanner availability per 100,000 population across four geographic regions (Northern, Southern, Eastern, and Western Europe). Total CT/MR volumes served as neuroimaging surrogates.

**Results:**

316 publications were identified (with none meeting predefined inclusion criteria). Eurostat data from 29 countries revealed substantial growth in imaging from 2015 to 2022. Per capita CT exam rates increased 40.8% (10,872 to 15,312 per 100,000 population), and MR scan rates increased 43.5% (5746 to 8244 per 100,000 population). Scanner availability also increased (CT scanners from 2.3 to 2.68, MR scanners from 1.43 to 2.11 per 100,000 population). Regional variations were evident: Western Europe showed the highest utilization rates, Eastern Europe demonstrated the largest relative growth despite lower absolute numbers. All regions experienced consistent growth except during the 2020 COVID-19 disruptions.

**Conclusion:**

Neuroimaging utilization has substantially increased across Europe from 2015 to 2022, with disproportionate growth in scan volumes relative to scanner availability. These findings highlight regional disparities in utilization and underscore the need for coordinated evidence-based appropriateness initiatives to support sustainable neuroimaging practice.

**Key Points:**

***Question*** *Have CT and MR neuroimaging utilization rates changed across Europe over the last decade compared to scanner availability?*

***Findings**** CT and MR scan rates increased 40.8% and 43.5%*,* respectively*,* from 2015 to 2022, reflecting increased per-scanner utilization across the continent.*

***Clinical relevance**** Neuroimaging examination volumes increased substantially across Europe from 2015 to 2022. This highlights the value of evidence-based imaging appropriateness initiatives to ensure sustainable healthcare resource utilization*.

**Graphical Abstract:**

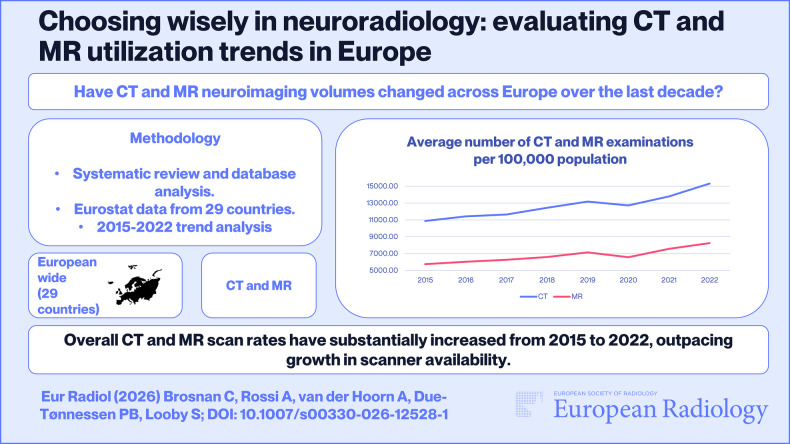

## Introduction

Neuroradiology has undergone a remarkable transformation over the last half-century. Once limited to rudimentary skull radiographs and invasive techniques such as myelography, pneumoencephalography and angiography, the field has been revolutionized since the advent of cross-sectional imaging, with the introduction of computed tomography (CT), developed by Godfrey Hounsfield in 1971 [1] and magnetic resonance imaging (MRI) soon after [2].

Since the introduction of CT and MR, scan utilization rates have consistently risen over the following decades across multiple healthcare systems worldwide. For example, Smith-Bindman et al demonstrated substantial increases in CT and MR scan rates in Washington state between 1997 and 2006 [3] and across seven US integrated health systems and Ontario between 2000 and 2016 [4]. Similar trends have been documented in Asia, where MR utilization has gradually increased since 1987 [5], and Australia, where emergency department CT rates nearly doubled between 2003 and 2015 [6].

Neuroimaging constitutes a substantial proportion of total CT and MR examinations, particularly for MR imaging, where brain and spine studies predominate. For example, in Norway, brain MR alone comprised 19% of all annual outpatient MR examinations (113,000/606,000 studies) on average between 2018 and 2022. This sustained increase in imaging utilization is reflected in the value of the medical imaging market, currently valued at $47.8 billion globally and projected to increase to almost $80 billion by 2034 [7], underscoring the growing clinical reliance on advanced imaging.

Continued growth in neuroimaging utilization raises several important questions regarding clinical appropriateness, resource stewardship and patient safety—all core concerns that align with the European Society of Neuroradiology’s Choosing Wisely initiative. Increased access to neuroimaging has the potential to improve diagnostic capabilities. However, increased utilization incurs direct costs for equipment, staffing and examination performance, increased radiation exposure from CT examinations and repercussions from incidental radiological findings [8–11]. This growth may also contribute to the phenomenon of “low-value imaging,” defined as imaging with little or no benefit [12], which can undermine the quality of care. More than 3.6 billion imaging examinations are estimated to be performed annually worldwide (11% of which are CT or MR), with 20–50% of these estimated to be “low-value” [13]. The challenge for modern neuroradiology is to balance the clinical benefits of advanced imaging with judicious use of resources and imaging appropriateness criteria.

Despite well-documented imaging trends in other regions, there remains a notable absence of recent studies specifically addressing neuroimaging utilization patterns across Europe. In light of this gap, we hypothesize that the volume and utilization of neuroimaging—specifically CT and MR—have also increased substantially in Europe over the last decade. This paper aims to systematically assess these trends using available international data sources.

## Materials and methods

This study combined a systematic search of published literature with descriptive analysis of data extracted from international health databases to assess neuroimaging trends in Europe over the last decade.

### Literature search

A systematic PubMed search was performed to identify any articles examining the overall volume of neuroimaging (specifically CT and MR) performed within Europe or any European country from January 2015 onwards. This was performed by two independent reviewers (C.B. and S.L.) in accordance with the Preferred reporting items for systematic reviews and meta-analyses (PRISMA 2020) statement [14] (Supplementary File 1). The search combined terms for neuroimaging modalities (“Neuroimaging,” “Neuro-imaging,” “Computed tomography,” “Magnetic resonance imaging,” etc.), utilization metrics (“Trend*,” “Utilis*,” “Volume,” “Imaging frequency” etc.) and names of all European countries using Boolean operators “AND” and “OR.” For the purposes of this study, “Europe” was defined according to the “United Nations geoscheme,” which includes a total of 44 countries in the European region [15]. Inclusion criteria were: (a) studies reporting the overall volume of neuroimaging (CT and/or MR), (b) performed in Europe or any European country, (c) published from January 2015, d) in English and e) involving human subjects. Case reports were excluded. Initially, titles and abstracts were screened. Full papers of suitable abstracts were then reviewed. A detailed breakdown of the search strategy used is detailed in Supplementary File 2.

### Analysis of international databases

Data on the total number of CT and MR scans performed per year across countries in Europe, as well as the number of CT and MR scanners per unit population, were obtained from the publicly available “Eurostat” and Organization for Economic Co-operation and Development (OECD) databases. Only countries with data available for both scan volume and scanner numbers were included for analysis. All data were standardized to rates per 100,000 population to facilitate cross-country comparisons. Countries were categorized into four geographic regions in accordance with the United Nations geo scheme: Northern Europe (Denmark, Estonia, Finland, Iceland, Latvia, Lithuania, Norway), Western Europe (Austria, Belgium, France, Germany, Liechtenstein, Luxembourg, Netherlands), Southern Europe (Croatia, Cyprus, Greece, Italy, Malta, North Macedonia, Serbia, Slovenia, Spain), Eastern Europe (Bulgaria, Czech Republic, Hungary, Poland, Romania, Slovakia) [15].

### Data analysis

All extracted data were analyzed descriptively, without formal trend or other inferential testing. This study used only published literature and publicly available data, and no ethical approval was required.

## Results

**Fig. 1:**
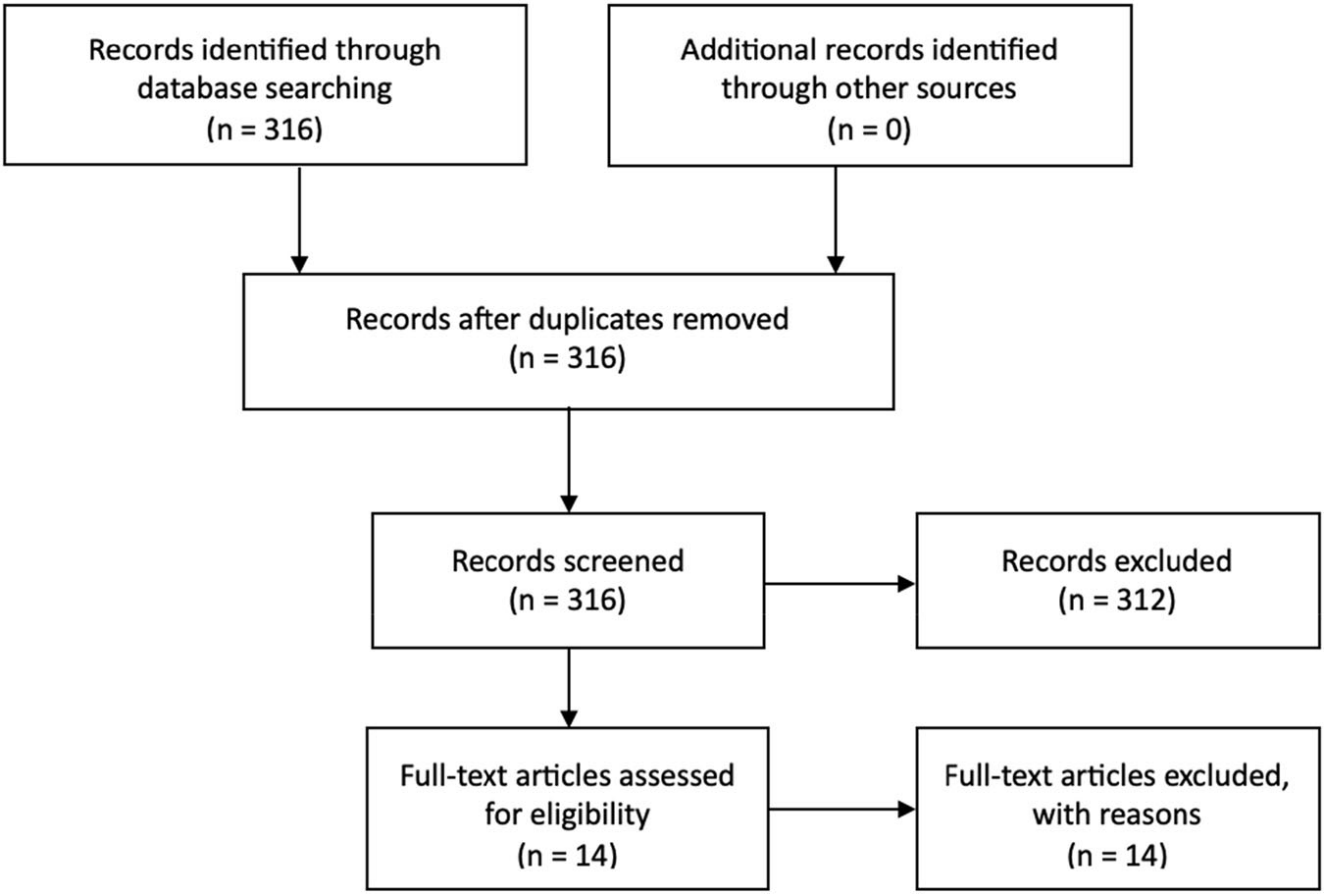
PRISMA flow diagram for included studies in systematic review

316 publications were found using the search criteria detailed above in “*Methods*.” 302 articles were removed following screening of titles and abstracts. Full papers were reviewed of the remaining 14 studies, with 0 studies deemed to have met predefined inclusion criteria. A PRISMA flow diagram is demonstrated in Fig. 1.

Eurostat was selected as the primary data source for analysis as it provided more comprehensive coverage, with scan volume data available for 29 countries from 2015 and scanner numbers for 31 countries from 2015. In comparison, the OECD database included scan volume data for 24 countries and scanner numbers for 31 countries. Data from OECD were cross-referenced against Eurostat for validation. There was complete concordance of data for the 24 countries present in both databases.

**Fig. 2:**
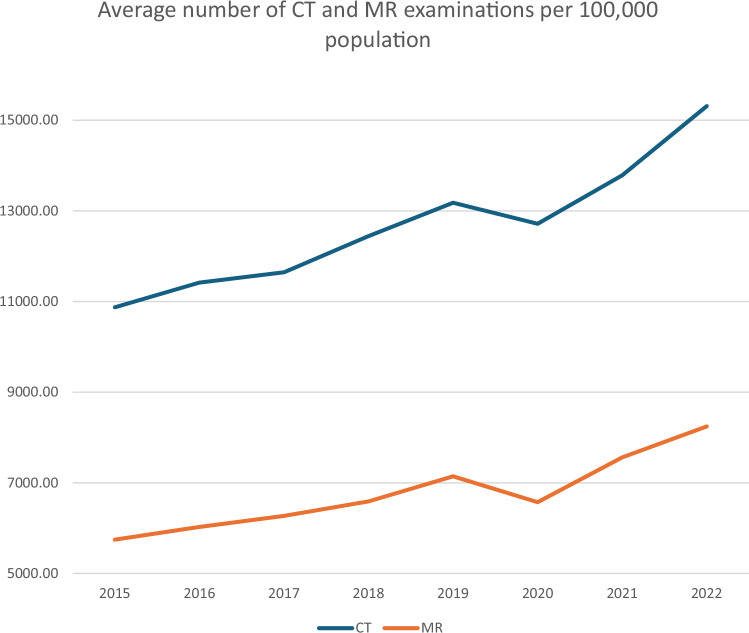
Average number of CT and MR examinations per 100,000 population across Europe (2015–2022)

**Fig. 3:**
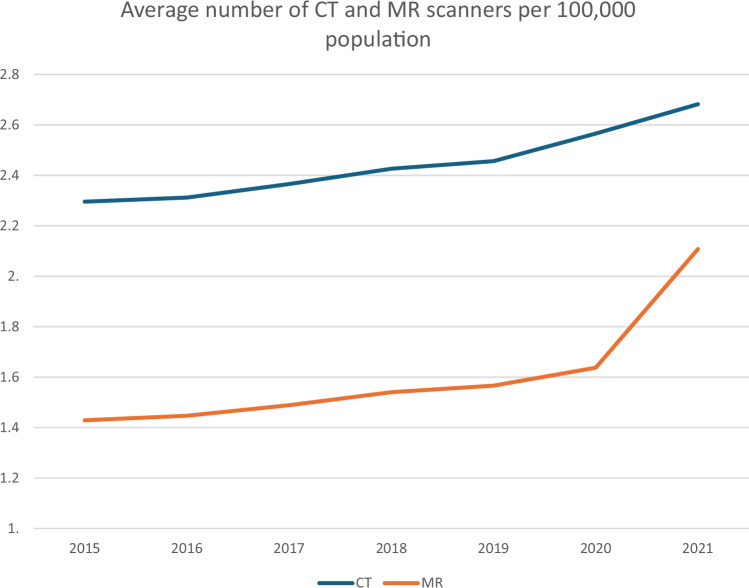
Average number of CT and MR scanners per 100,000 population across Europe (2015–2021)

Across Europe, the average number of CT scans performed per 100,000 population increased by 40.8% from 10,872 in 2015 to 15,312 in 2022, and the average number of MR scans performed per 100,000 population increased by 43.5% from 5746 in 2015 to 8244 in 2022. The total number of scans increased each year over this time, except during 2020, with a drop in the number of scans secondary to the global COVID-19 pandemic. The average number of CT scanners per 100,000 population increased by 16.5% from 2.3 in 2015 to 2.68 in 2021, and the average number of MR scanners per 100,000 population increased by 47.6% from 1.43 per 100,000 population in 2015 to 2.11 per 100,000 population in 2021. The total number of CT and MR scanners increased every year. A full breakdown of the number of CT and MR scans and scanners is provided in Tables 1–4, along with representative temporal line graphs in Figs. 2 and 3.

**Table 1:** CT examinations per 100,000 population (2015–2022)

Country	2015	2016	2017	2018	2019	2020	2021	2022
Austria	17,512	17,256	17,336	18,356	19,619	18,200	19,909	21,259
Belgium	19,845	19,967	20,054	20,190	20,487	20,504	22,325	22,512
Bulgaria	5137	6025	6575	7460	7808	7370	8553	9906
Cyprus	13,995	13,157	14,137	15,029	13,281	9289	7237	7905
Czech republic	10,191	10,744	10,355	11,086	11,411	10,911	11,990	12,468
Germany	14,310	14,845	13,989	14,470	15,119	14,999	15,979	16,177
Denmark	16,179	16,066	17,283	18,464	19,017	12,000	12,789	12,884
Estonia	12,717	13,798	13,814	13,351	13,621	12,761	14,384	14,594
Greece	14,601	15,033	19,454	21,389	19,543	12,191	15,036	18,628
Spain	10,487	10,966	11,495	11,810	12,433	11,340	13,398	14,143
Finland	3908	4170	5403	5750	6721	4121	4592	6422
France	18,870	19,402	18,958	19,516	19,864	19,746	21,752	22,321
Croatia	7839	8558	9466	10,404	11,573	10,533	13,746	12,693
Hungary	-	-	-	17,626	18,551	17,564	20,441	22,142
Iceland	19,095	20,480	21,374	22,727	23,209	21,541	-	-
Italy	8844	8578	8989	9402	9727	8750	10191	10695
Liechtenstein	4195	4391	3841	4108	4559	8871	11212	11590
Lithuania	9476	10,327	10,632	12,090	13,255	11,075	14,867	16,224
Luxembourg	18,796	19,118	19,124	19,720	20,119	19,058	22,019	22,372
Latvia	16,851	17,836	17,194	18,076	18,420	19,150	21,782	24,469
North Macedonia	2560	2897	3060	3207	3593	2738	3843	-
Malta	8061	8964	8701	9725	10,221	10,495	11,485	11,056
Netherlands	8081	8866	9641	10,526	11,092	11,395	13346	13,219
Norway	7408	7730	8038	8313	8593	8473	9054	9284
Poland	7015	7633	8146	8541	10,062	9086	11,896	13,434
Romania	2593	2738	2882	3374	3403	3642	5804	6984
Serbia	4458	4737	4879	5182	5932	5225	7058	9240
Slovenia	6184	6510	7089	7602	8396	8431	9989	10,696
Slovakia	15,620	16,227	1539	15,515	16,014	14,368	16,705	17,516

**Table 2:** MR examinations per 100,000 population (2015–2022)

Country	2015	2016	2017	2018	2019	2020	2021	2022
Austria	11,743	12,024	13,070	14,139	14,795	14,051	15,960	16,360
Belgium	8556	8940	9394	9540	9812	8742	10,419	10,918
Bulgaria	633	839	1012	1249	1326	1058	1187	1903
Cyprus	687	695	660	570	635	580	8956	9672
Czech republic	4796	4980	5269	5472	6009	5822	6606	7029
Germany	13,860	14,344	14,075	14,508	14,983	14,995	15,769	15,565
Denmark	8210	8220	8696	8910	9064	6973	7529	7388
Estonia	4679	4988	4852	5034	5081	5050	5585	5719
Greece	6053	6403	7317	8338	8739	4646	10660	10684
Spain	7833	8324	8855	9239	9910	8476	10974	11338
Finland	4170	4150	4698	4954	5307	3969	4312	4468
France	10,264	11,073	11,402	11,921	12,283	11,720	13,620	14,334
Croatia	4008	4736	4794	5192	5971	5533	6932	7672
Hungary	-	-	-	5130	5419	4474	4979	5591
Iceland	8190	9292	9095	10,276	10,825	10,387	-	-
Italy	7796	6747	7136	7403	7540	6470	7724	8054
Liechtenstein	10,660	10,767	9209	9021	8695	9709	11,401	12,582
Lithuania	4078	4506	5009	5749	6698	5420	7946	8715
Luxembourg	7453	7566	7315	7495	8136	8391	10,482	10,555
Latvia	4257	4768	5535	6461	6923	7153	8249	8779
North Macedonia	963	1047	1054	1079	1333	681	956	-
Malta	4981	5347	5339	5626	6428	5259	8148	7788
Netherlands	5176	4884	5110	5215	5946	5860	5805	5723
Norway	10,572	10,623	11,246	11,589	11,656	11,754	13,283	14,080
Poland	2782	2981	3447	3724	4221	4098	5394	6078
Romania	1022	1083	1442	1380	1416	1444	2380	3263
Serbia	1363	1468	1344	1330	1577	1302	1593	2030
Slovenia	4236	5189	6177	6974	8007	7952	9712	11,376
Slovakia	5677	6143	6296	6952	7367	6842	7607	7368

**Table 3:** CT scanners per 100,000 population (2015–2021)

Country	2015	2016	2017	2018	2019	2020	2021
Belgium	2.36	2.39	2.38	2.39	2.41	2.40	2.46
Bulgaria	3.36	3.47	3.58	3.89	3.94	4.05	-
Czechia	1.61	1.55	1.58	1.61	1.64	1.63	-
Denmark	3.77	3.91	3.97	3.97	4.06	4.06	4.37
Germany	3.51	3.52	3.51	3.53	-	-	-
Estonia	1.75	1.75	1.82	1.89	1.88	2.03	-
Ireland	1.77	1.72	1.91	2.03	2.13	2.03	2.04
Greece	3.61	3.59	3.42	4.06	4.25	4.37	-
Spain	1.80	1.83	1.87	1.91	1.92	2.00	-
France	1.66	1.70	1.73	1.76	1.82	1.90	-
Croatia	1.52	1.77	1.82	1.96	1.99	2.22	-
Italy	3.33	3.43	3.46	3.53	3.64	3.75	4.00
Cyprus	3.42	3.41	3.37	3.33	3.51	3.81	-
Latvia	3.69	3.62	3.91	3.84	3.71	3.74	-
Lithuania	2.10	2.30	2.33	2.43	2.65	3.11	2.93
Luxembourg	1.76	1.71	1.68	1.64	1.61	2.22	2.21
Hungary	0.84	0.89	0.92	0.94	0.94	0.96	-
Malta	1.80	1.98	1.92	1.86	1.98	1.94	-
Netherlands	1.38	1.30	1.35	1.42	1.49	1.47	-
Austria	2.89	2.91	2.86	2.88	2.87	2.85	-
Poland	1.72	1.73	1.69	1.81	1.82	2.01	-
Romania	1.18	1.26	1.40	1.59	1.75	1.91	-
Slovenia	1.31	1.40	1.50	1.59	1.82	1.90	1.90
Slovakia	1.79	1.73	1.73	1.84	1.78	1.91	-
Finland	2.15	2.42	2.45	1.65	1.63	1.70	-
Iceland	3.93	3.88	4.37	4.82	4.71	4.64	-
Liechtenstein	2.67	2.65	2.63	2.61	2.59	2.57	2.56
Norway	-	1.55	1.71	1.94	2.64	3.10	3.00
Switzerland	3.77	3.89	3.93	3.88	3.87	3.96	-
Serbia	0.99	0.96	1.05	1.12	1.17	1.23	1.35
Türkiye	1.43	1.45	1.48	1.49	1.47	1.50	-

**Table 4:** MR scanners per 100,000 population (2015–2021)

Country	2015	2016	2017	2018	2019	2020	2021
Belgium	1.17	1.16	1.16	1.16	1.15	1.14	1.14
Bulgaria	0.71	0.79	0.99	1.04	1.13	1.15	-
Czechia	0.83	0.85	0.94	1.03	1.04	1.10	-
Germany	3.36	3.45	3.47	3.45	-	-	-
Estonia	1.22	1.37	1.37	1.36	1.43	1.50	
Ireland	1.40	1.47	1.52	1.60	-	-	-
Greece	2.48	2.69	2.65	2.93	3.19	3.35	-
Spain	1.58	1.61	1.64	1.72	1.76	1.82	-
France	1.26	1.35	1.42	1.47	1.54	1.63	-
Croatia	1.09	1.08	1.14	1.25	1.25	1.53	-
Italy	2.82	2.84	2.87	2.88	3.02	3.12	3.42
Cyprus	1.89	2.00	2.09	2.07	2.04	2.02	-
Latvia	1.26	1.38	1.39	1.35	1.52	1.58	-
Lithuania	1.10	1.22	1.24	1.25	1.40	1.43	-
Luxembourg	1.23	1.20	1.17	1.15	1.45	1.74	1.73
Hungary	0.36	0.40	0.47	0.49	0.49	0.49	-
Malta	1.12	1.10	1.07	1.03	1.19	1.16	-
Netherlands	1.25	1.28	1.30	1.31	1.38	1.34	-
Austria	2.07	2.24	2.30	2.35	2.50	2.53	-
Poland	0.76	0.79	0.79	0.92	0.93	1.05	-
Romania	0.54	0.59	0.71	0.90	1.06	1.18	-
Slovenia	0.92	1.11	1.16	1.21	1.24	1.33	1.33
Slovakia	0.88	0.90	0.96	0.95	0.95	0.99	-
Finland	2.59	2.55	2.71	2.74	2.88	3.06	3.09
Iceland	2.12	2.09	2.04	1.98	1.94	1.91	-
Liechtenstein	2.67	2.65	2.63	2.61	2.59	2.57	2.56
Norway	-	0.44	0.53	0.96	1.74	1.90	3.12
Serbia	0.31	0.31	0.34	0.39	0.39	0.46	0.47
Türkiye	1.02	1.05	1.10	1.12	1.09	1.13	-

In Western Europe, the average number of CT scans performed per 100,000 population increased by 27.4% from 14,517 in 2015 to 18,493 in 2022. The average number of MR scans per 100,000 population increased by 27.1% from 9673 in 2015 to 12,291 in 2022. Over the same period, the average number of CT scanners per 100,000 population increased by 3.9% from 2.32 in 2015 to 2.41 in 2021, while the average number of MR scanners per 100,000 population decreased by 2.2% from 1.85 in 2015 to 1.81 in 2021.

In Northern Europe, the average number of CT scans performed per 100,000 population increased by 14.3% from 12,234 in 2015 to 13,980 in 2022. The average number of MR scans per 100,000 population increased by 29.9% from 6308 in 2015 to 8191 in 2022. The average number of CT scanners per 100,000 population increased by 35.2% from 2.73 in 2015 to 3.69 in 2021, while the average number of MR scanners per 100,000 population increased by 87.3% from 1.66 in 2015 to 3.11 in 2021.

In Southern Europe, the average number of CT scans performed per 100,000 population increased by 38.8% from 8559 in 2015 to 11,882 in 2022. The average number of MR scans per 100,000 population increased by 103.6% from 4,213 in 2015 to 8577 in 2022. The average number of CT scanners per 100,000 population increased by 9% from 2.22 in 2015 to 2.42 in 2021, while the average number of MR scanners per 100,000 population increased by 13.7% from 1.53 in 2015 to 1.74 in 2021.

In Eastern Europe, the average number of CT scans performed per 100,000 population increased by 69.4% from 8111 in 2015 to 13,742 in 2022. The average number of MR scans per 100,000 population increased by 74.5% from 2982 in 2015 to 5205 in 2022. The average number of CT scanners per 100,000 population increased by 18.9% from 1.75 in 2015 to 2.08 in 2021, while the average number of MR scanners per 100,000 population increased by 36.8% from 0.68 in 2015 to 0.93 in 2020.

## Discussion

Statistics obtained from Eurostat and OECD demonstrate consistent and substantial growth in the utilization of CT and MR across Europe over the last decade. Specifically, per capita CT exam rates increased from 10,872 to 15,312 per 100,000 population, while MR scan rates increased from 5746 to 8244 per 100,000 from 2015 to 2022. This data suggests sustained growth in neuroimaging across the continent, with the rate of scans being performed outstripping the increase in the number of scanners. This is particularly apparent in CT imaging, with an increase in scan volume of 40.8% since 2015, coupled with an increase in scanner numbers of only 16.5%. Whilst regional disparities in the total number of scans performed within Europe were observed, areas with lower scan utilization were seen to experience significantly higher rates of growth.

These findings align with increasing neuroimaging volumes documented across multiple healthcare systems globally, with dramatic rises and consistent increases demonstrated throughout the United States since the late 1990s onwards [16–18], consistent increases in volume within Asia since 1987 [5] and evidence of significant increases in volumes within East Asia more recently from 2020 [19]. This convergent pattern across diverse healthcare systems and economic contexts suggests that drivers of increased neuroimaging utilization transcend regional boundaries and healthcare delivery models. Multiple mechanisms may contribute to increased neuroimaging utilization.

Advancing management paradigms for multiple neurological conditions has contributed to increased volumes. Evolving clinical practice guidelines increasingly incorporate advanced neuroimaging techniques, including stroke management protocols (with routine integration of multimodal imaging, including perfusion imaging [20]), intensified tumor surveillance criteria such as RANO 2.0 [21] and advanced imaging techniques such as MR perfusion being advocated for integration into assessment of treatment response in tumors [22, 23], and expanded multiple sclerosis monitoring recommendations [24].

Demographic shifts toward aging populations may also influence demand, as CNS pathology incidence increases with age [25–27]. Currently, 21.6% of the European Union population is 65 or over, with this figure projected to increase [28]. These trends would predict continued growth in neuroimaging utilization.

Technological advancements, with continual improvements in CT and MR imaging speed, accessibility and image quality have enhanced diagnostic capabilities [29, 30]. Whilst these developments enabling earlier, more accurate diagnoses represent appropriate utilization drivers, increased accessibility may lower barriers to ordering examinations. A recent audit performed by Singer et al documented CT appropriateness rates ranging from 58% to 86% across 7 European countries when using standardized imaging guidance, with stricter guidance adherence in areas where cost constraints are greatest [31]. Beyond clinical factors, defensive medicine practices also contribute to utilization patterns, with 61 and 64% of surveyed neurosurgeons across Turkey and the Netherlands admitting to ordering imaging studies solely for defensive purposes [32, 33], with a similar study in the US reporting 72% of respondents admitting to similar practices [34].

Scan utilization rates are rising faster than equipment growth. Without commensurate workforce and infrastructure expansion, disproportionate volume growth may contribute to scheduling pressures, extended waiting times and increased radiologist workload, with documented burnout prevalence in radiology already ranging from 33 to 88% [35].

Increased imaging volumes have important implications at the patient, system and society levels. CT use contributes to cumulative population radiation exposure, with recent estimates suggesting that contemporary practice in the United States may be associated with a substantial future cancer burden [36]. Incidental findings are common, with reported frequencies ranging from 15.8–31.3% [9], and can generate anxiety [8, 11], additional testing and procedure-related complications [10]. At the system level, a considerable proportion of examinations worldwide are classified as “low-value” [13], with neuroimaging featuring prominently among commonly cited unnecessary services [37]. Reducing such services could create substantial savings and provide resources for high-value care [37]. Imaging activity also carries an environmental footprint, with radiology contributing to global greenhouse gas emissions and neuroradiology accounting for a substantial share of activity, alongside broader sustainability concerns related to finite resources and contrast media [38, 39].

Several limitations warrant acknowledgment. Published literature and international databases lack neuroimaging-specific volume data. As a result, this study lacks quantitative data specific to neurological studies and uses overall CT/MR scans and scanners as a surrogate marker for neuroimaging trends. No data were provided regarding clinical appropriateness or outcomes associated with increased imaging utilization, precluding any distinction between necessary and potentially low-value examinations. European countries also demonstrate substantial heterogeneity in healthcare delivery models and clinical pathways that may influence utilization patterns independent of clinical need. This study cannot account for such system-level confounders. UK data were unavailable in Eurostat and OECD during the study period. While alternative sources exist [40], potential methodological differences in data collection and reporting precluded inclusion. However, available UK statistics suggest patterns similar to those of other Western European countries. Administrative mechanisms such as changes in coding practices and billing systems may also contribute to increased examination documentation independent of actual clinical activity. Distinguishing genuine increases in utilization from improved administrative capture represents an important consideration.

Our findings support the relevance of evidence-based appropriateness initiatives to optimize utilization within resource constraints. Existing European resources, including the ESR iGuide and RCR iRefer [41, 42], provide specialty-specific appropriateness recommendations. Future initiatives should harmonize these platforms. Initiatives such as the American College of Radiology (ACR) Appropriateness Criteria [43] and the Choosing Wisely campaign, launched by the American Board of Internal Medicine Foundation in 2012, have previously demonstrated a substantial impact on neuroimaging in the US and would help to serve as a valuable model for future European initiatives [16, 44]. Awareness amongst clinicians outside of radiology regarding any newly released guidelines would be paramount to address inappropriate exam requests.

This analysis demonstrates substantial growth in the utilization of neuroimaging across Europe over the last decade, with a more modest increase in the number of scanners across the continent. Findings emphasize a growing need for coordinated European initiatives to optimize the use of neuroimaging. Proposed solutions include the development of integrated and readily available appropriateness criteria for common presentations to help manage growing demand while maintaining high-quality patient care. Future research should focus on developing Europe-specific imaging guidelines and monitoring clinical outcomes associated with the varying utilization patterns across regions to ensure that increased neuroimaging access translates to improved patient outcomes rather than simply increased strain on resources and healthcare costs.

## Supplementary information

Supplementary information

## Abbreviations

CT: Computed tomography
MRI: Magnetic resonance imaging
OECD: Organization for Economic Co-operation and Development
